# Hydrogen Mitigated Doxorubicin-Induced Liver Injury via Nrf2/HO-1 Pathway Activation

**DOI:** 10.3390/ijms27062774

**Published:** 2026-03-19

**Authors:** Meng-Fan Sun, Ji-Xian Song, Miao Tang, Bo-Han Yu, Yao Xiao, Yu-Hui Gao, Zi-Xuan Yao, Ke-Ying An, Zhen-Qun Zhang, Yong-Qing Shen, Ya-Shuo Zhao

**Affiliations:** 1 Hebei Technology Innovation Center of TCM Combined Hydrogen Medicine, Hebei University of Chinese Medicine, Shijiazhuang 050200, China; 2 Transformation Center for Scientific and Technological Achievements, Hebei University of Chinese Medicine, Shijiazhuang 050200, China; 3 School of Nursing, Hebei University of Chinese Medicine, Shijiazhuang 050200, China

**Keywords:** doxorubicin, liver injury, oxidative stress, Nrf2

## Abstract

Drug-induced liver injury constitutes a major concern within the spectrum of drug-related pathologies. The precise mechanisms underlying doxorubicin (DOX)-induced liver injury remain inadequately elucidated. Hydrogen is known for its selective antioxidant properties and favorable safety profile; however, its protective effects against DOX-induced liver injury have not been fully clarified. In this study, a model of DOX-induced liver injury was established to evaluate hepatic function and pathological alteration, thereby assessing the therapeutic efficacy of hydrogen. Further investigations were conducted to quantify oxidative stress and inflammatory markers to elucidate the potential mechanisms involved. Hydrogen treatment significantly mitigated DOX-induced liver damage and inhibited hepatocyte fibrosis. Hydrogen was found to suppress apoptosis, reduce oxidative stress levels, and ameliorate inflammatory responses in the liver tissue of DOX mice. The protective effect was predominantly facilitated by the modulation of the Nrf2/HO-1 pathway. Importantly, the hepatoprotective effect of hydrogen was negated following the administration of an Nrf2 inhibitor in HepG2 cells. These results suggest that hydrogen may mitigate DOX-induced liver injury by activating the Nrf2/HO-1 signaling pathway, consequently diminishing oxidative stress and inflammatory responses.

## 1. Introduction

Annually, approximately 70,000 individuals succumb to malignant neoplasms [1]. Although antineoplastic agents have the potential to enhance survival rates among cancer patients, their use is frequently associated with significant adverse effects, including hepatotoxicity and nephrotoxicity [2,3]. The liver, as the primary organ responsible for drug metabolism and detoxification, is particularly vulnerable to toxicity induced by antineoplastic drugs [4]. Consequently, it is imperative to address and mitigate the hepatic toxicity associated with these anti-tumor therapies. Implementing such strategies not only improves the safety of treatment but also facilitates the administration of effective anticancer regimens without compromising liver function.

Doxorubicin (DOX), an anthracycline, is recognized as one of the most clinically effective and widely utilized antitumor agents [5]. Nevertheless, its clinical application is often constrained by significant toxicity. The most prevalent adverse effects of DOX therapy include hematopoietic failure, cardiotoxicity, and hepatic damage, with approximately 40% of patients undergoing DOX treatment experiencing liver damage [6]. The principal molecular mechanism responsible for this toxicity involves the generation of reactive oxygen species (ROS) during the hepatic metabolism of DOX, resulting in oxidative stress, decreased levels of antioxidant enzymes, apoptosis, inflammation, and mitochondrial dysfunction [7,8]. Therefore, targeting oxidative stress regulation is essential for mitigating DOX-induced hepatic damage. Natural antioxidants and phytochemicals, such as those present in plant extracts, have shown protective effects by scavenging free radicals, enhancing antioxidant defenses, and modulating inflammatory responses [9]. For example, taxifolin (TAX) has been reported to mitigate chemotherapy-induced hepatotoxicity by inhibiting ferroptosis and oxidative pathways, underscoring the potential of adjunct therapies to reduce DOX-related liver injury while preserving its anticancer efficacy [10]. Consequently, addressing the toxicity associated with DOX-based regimens is crucial for enhancing patient outcomes and ensuring safer administration.

Hydrogen has emerged as a promising therapeutic agent due to its selective antioxidative properties, non-toxicity to cells, and excellent biocompatibility [11]. Numerous studies have demonstrated that hydrogen could alleviate DOX-induced cardiotoxicity by inhibiting apoptosis, reducing ferroptosis, and enhancing autophagy [12,13,14]. DOX-induced hepatotoxicity involves mechanisms such as oxidative stress, mitochondrial dysfunction, and inflammatory responses, which are mechanisms similar to those observed with other chemotherapeutic agents like cisplatin [15,16]. However, it remains uncertain whether hydrogen could mitigate DOX-induced liver toxicity and the specific mechanisms involved. Therefore, this study aims to establish in vivo and in vitro models of DOX-induced liver injury to investigate the protective effects of hydrogen and elucidate its potential mechanisms.

## 2. Results

### 2.1. Hydrogen Attenuated the Liver Dysfunction Induced by DOX

**Figure 1:**
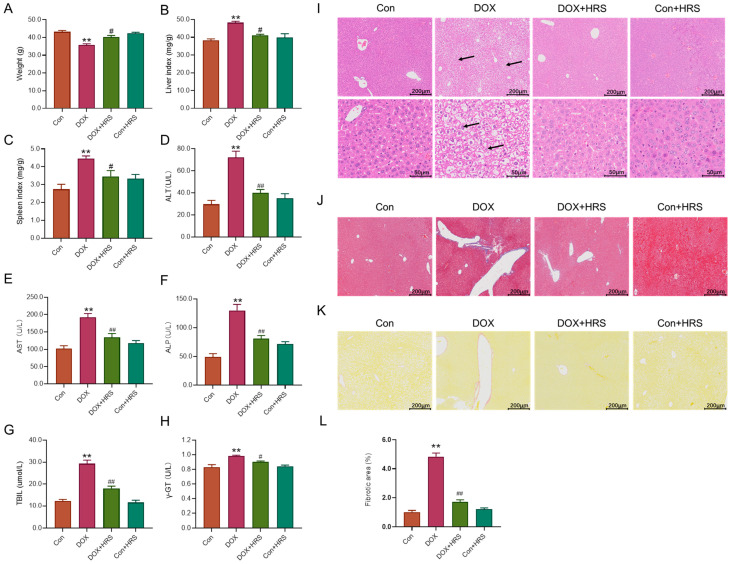
Hydrogen attenuated the liver dysfunction induced by DOX. (**A**) The body weights of the mice from different groups (n = 6). (**B**,**C**) The liver and spleen index of the mice from different groups (n = 6). (**D**–**H**) The serum content of ALT, AST, ALP, TBIL, and γ-GT of the mice from different groups (n = 6). (**I**) The HE staining in liver tissue (scale bar = 200 μm or 50 μm, n = 3). (Arrows point to necrotic hepatocytes.). (**J**) The Masson staining in liver tissue (scale bar = 200 μm, n = 3). (**K**) The SiriusRed staining in liver tissue (scale bar = 200 μm, n = 3). (**L**) The quantification of the fibrotic area in liver tissue. The data are presented as the means ± SEM. ** *p* < 0.01 vs. Con group. *^#^ p* < 0.05, *^##^ p* < 0.01 vs. DOX group.

Initially, we evaluated the baseline condition of mice subjected to DOX treatment. As shown in Figure 1A–C, the DOX-treated mice exhibited reduced body weight and increased liver and spleen indices compared to the control group. However, these parameters were significantly ameliorated following hydrogen-rich saline solution (HRS) treatment in the DOX group (Figure 1A–C). Subsequently, we assessed liver function-related biochemical markers. The serum levels of alanine transaminase (ALT), aspartate transaminase (AST), alkaline phosphatase (ALP), total bilirubin (TBIL), and γ-glutamyl transferase (γ-GT) in DOX-treated mice were significantly elevated compared to normal levels (Figure 1D–H). Concurrently, pathological staining revealed disordered liver lobules in the DOX group, characterized by extensive necrotic liver cells around the portal area, along with inflammatory factor infiltration and fibrosis (Figure 1J–L). These findings indicated hepatic dysfunction in DOX-treated mice. Moreover, HRS administration resulted in decreased levels of ALT, AST, ALP, TBIL, and γ-GT, and improved the pathological abnormalities in the liver structure of DOX-treated mice (Figure 1D–L).

### 2.2. Analysis of the Liver Injury Targets of DOX Through the GEO Database

**Figure 2:**
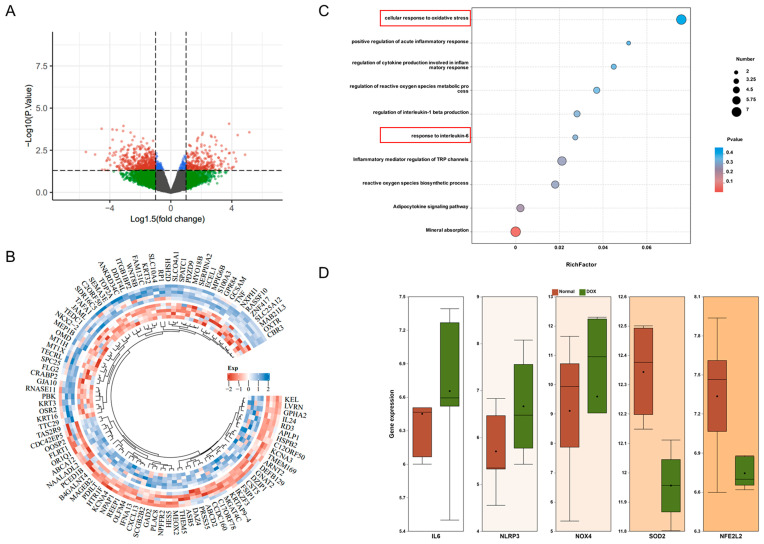
Possible target screening by GEO analysis. (**A**) Volcano plot of differentially expressed genes (DEGs) between the Con and DOX groups. (Red: significantly upregulated genes. Green: significantly downregulated genes. Blue: genes with extreme fold change but non-significant *p*-value. Grey: non-significant genes). (**B**) Circular plot of gene ontology (GO) analysis of cell component (CC), molecular function (MF), and biological process (BP). (**C**) Bubble plot of Kyoto Encyclopedia of Genes and Genomes (KEGG) enrichment analysis. (**D**) Box plots of *IL-6*, *NLRP3*, *NOX4*, *SOD2*, and *Nfe2l2* gene levels.

Through Gene Expression Omnibus (GEO) data, we obtained gene expression-related information from HepG2 cells subjected to DOX treatment. Subsequent analysis identified several genes exhibiting altered expression post-DOX treatment (Figure 2A,B). Enrichment analysis indicated that these genes were predominantly linked to oxidative stress and inflammatory responses (Figure 2C). A detailed analysis revealed that the expression levels of interleukin-6 (*IL-6*), NOD-like receptor protein 3 (*NLRP3*), nicotinamide adenine dinucleotide phosphate oxidase 4 (*NOX4*), manganese superoxide dismutase (*SOD2*), and nuclear factor erythroid-derived 2-like 2 (*NFE2L2*) genes were upregulated following DOX treatment (Figure 2D).

### 2.3. Hydrogen Attenuated Apoptosis in the Liver of DOX-Induced Mice

**Figure 3:**
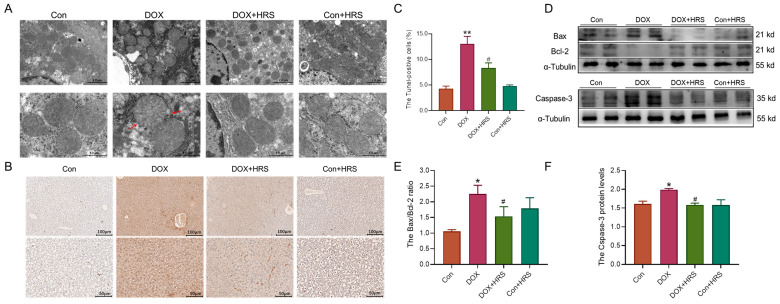
Hydrogen decreased apoptosis in the liver of DOX-induced mice. (**A**) TEM images of mitochondria in liver tissue (scale bar  =  2.0 μm or 1.0 μm, n = 3). (The red arrows indicate disrupted mitochondrial cristae). (**B**,**C**) Tunel staining in liver tissue (scale bar  =  100 μm or 50 μm, n =  3). (**D**–**F**) Expression and statistics of Bax/Bcl-2 and Caspase-3 protein levels in liver tissue (n = 4). The results are presented as the mean ± SEM. * *p* < 0.05, ** *p* < 0.01 vs. Con group. ^#^ *p* < 0.05 vs. DOX group.

Subsequently, we investigated apoptosis in the liver tissue of DOX-treated mice. Transmission electron microscope (TEM) images revealed significant impairment and structural incompleteness in the mitochondrial cristae of the liver in DOX-treated mice compared to normal mice (Figure 3A). Tunel staining indicated that, relative to the control group, there was a substantial aggregation of positive apoptotic cells in the liver injury regions of the DOX group. Treatment with HRS ameliorated the apoptosis in the liver induced by DOX (Figure 3B,C). Furthermore, western blot analysis revealed an increased Bax/Bcl-2 ratio and elevated levels of caspase-3 in the DOX group. These elevated levels were reduced following HRS administration in DOX-treated mice (Figure 3D–F). Collectively, these findings indicated that HRS effectively mitigated mitochondrial damage and cell apoptosis induced by DOX in liver tissue.

### 2.4. Hydrogen Efficiently Inhibited Oxidative Stress in Liver Tissue Induced by DOX

**Figure 4:**
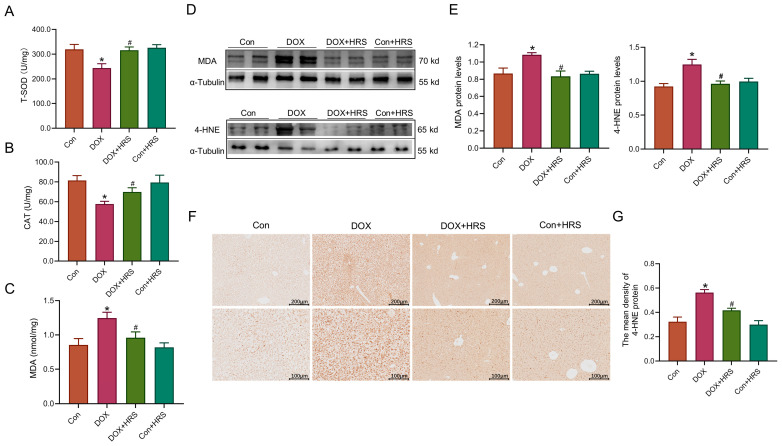
Hydrogen efficiently inhibited oxidative stress in liver tissue induced by DOX. (**A**,**B**) The activities of T-SOD and CAT in liver tissue (n = 6). (**C**) The MDA content in the liver tissues (n = 6). (**D**,**E**) The expression and statistics of MDA and 4-HNE protein levels measured by Western blot (n = 4). (**F**,**G**) The expression and statistics of 4-HNE protein measured by IHC staining (scale bar  =  200 μm or 100 μm, n =  3). The results are presented as the mean ± SEM. * *p* < 0.05 vs. Con group. ^#^ *p* < 0.05 vs. DOX group.

We conducted a simultaneous evaluation of oxidative stress levels in the liver. Resluts demonstrated a significant reduction in the activities of total superoxide dismutase (T-SOD) and catalase (CAT) in the livers of mice treated with DOX, as shown in Figure 4A,B. Furthermore, we measured the levels of lipid peroxidation products, specifically malondialdehyde (MDA) and 4-Hydroxynonenal (4-HNE). The MDA content was found to be elevated in the liver tissue of DOX-treated mice (Figure 4C), and a concurrent increase in MDA protein levels was observed (Figure 4D). Additionally, the protein level of 4-HNE was elevated in the livers of DOX-treated mice, as determined by immunohistochemical staining (IHC) and western blot analysis (Figure 4E–G). Importantly, HRS treatment resulted in a significant enhancement of antioxidant levels (T-SOD and CAT) and a reduction in the levels of lipid peroxidation products (MDA and 4-HNE).

### 2.5. Hydrogen Efficiently Inhibited Inflammation in Liver Tissue Induced by DOX

**Figure 5:**
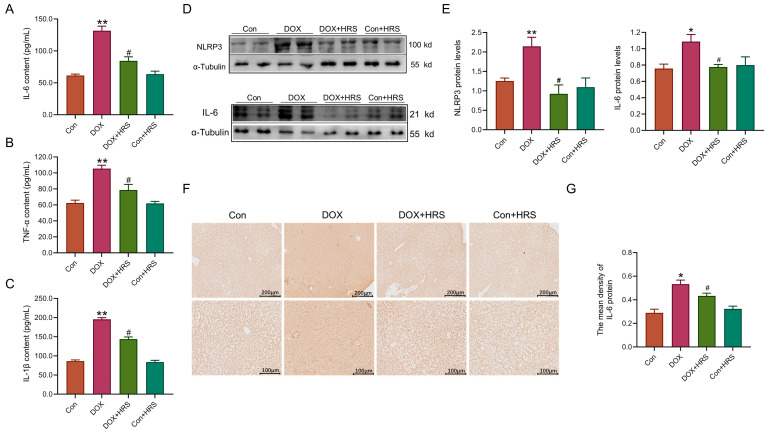
Hydrogen markedly declined inflammation induced by DOX. (**A**–**C**) The content of IL-6, IL-1β, and TNF-α in serum, measured by ELISA (n = 6). (**D**,**E**) The expression and statistics of NLRP3 and IL-6 protein levels measured by Western blot (n = 4). (**F**,**G**) The expression and statistics of IL-6 protein measured by IHC staining (scale bar  =  200 μm or 100 μm, n =  3). The results are presented as the mean ± SEM. * *p* < 0.05, ** *p* < 0.01 vs. Con group. ^#^ *p*< 0.05 vs. DOX group.

We analyzed pro-inflammatory factors in mice treated with DOX. Enzyme-linked immunosorbent assay (ELISA) results showed that the levels of pro-inflammatory cytokines IL-6, interleukin-1β (IL-1β), and tumor necrosis factor α (TNF-α) were significantly higher in the serum of DOX mice (Figure 5A–C). After HRS treatment, these levels significantly dropped (Figure 5A–C). At the same time, the expression of the NLRP3 inflammasome protein was notably higher in the livers of DOX-treated mice (Figure 5D,E). HRS treatment led to a significant decrease in NLRP3 protein levels (Figure 5D,E). Western blot and IHC results indicated that IL-6 protein levels were significantly elevated in the liver tissue of DOX-treated mice, which decreased after HRS treatment (Figure 5D–G). These results suggested that HRS effectively reduced the activation of pro-inflammatory factors caused by DOX.

### 2.6. Hydrogen Activated the Nrf2/HO-1 Pathway to Attenuate Liver Injury in DOX Mice

**Figure 6:**
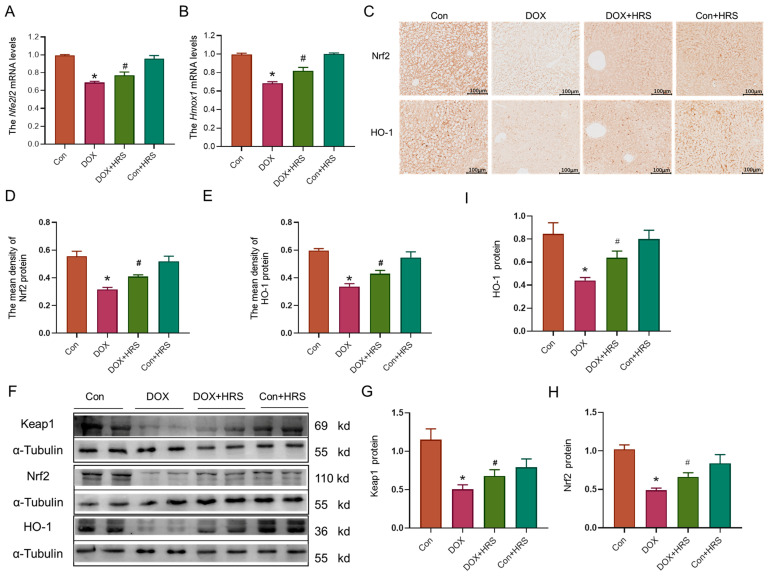
Hydrogen activated the Nrf2/HO-1 pathway to attenuate liver injury in DOX mice. (**A**,**B**) Nfe2l2 and Hmox1 gene expression in liver tissue (n = 3). (**C**–**E**) IHC staining and statistics of Nrf2 and HO-1 protein (scale bar = 100 µm, n = 3). (**F**–**I**) Expression and statistics of Keap1, Nrf2, and HO-1 protein levels measured by Western blot (n = 4). The results are presented as the mean ± SEM. * *p* < 0.05 vs. Con group. ^#^ *p*< 0.05 vs. DOX group.

Subsequently, we investigated the Nrf2/HO-1 signaling pathway in the liver tissue of DOX-treated mice. Q-PCR analysis indicated a reduction in the mRNA expression levels of *Nfe2l2* (Nrf2 gene) and *Hmox1* (heme oxygenase-1, HO-1 gene) in the liver tissues of DOX mice (Figure 6A,B). IHC results demonstrated a significant decrease in the protein expression of Nrf2 and HO-1 in the liver tissue of DOX-treated mice (Figure 6C–E). Furthermore, western blot analysis corroborated these findings by showing a corresponding reduction in the protein levels of Keap1, Nrf2, and HO-1 (Figure 6F–I). Notably, following HRS treatment, the previously diminished Nrf2 and HO-1 gene or protein levels exhibited varying degrees of recovery.

### 2.7. Involvement of the Nrf2/HO-1 Pathway in Hydrogen-Mediated Protection in HepG2 Cells Treated by DOX

**Figure 7:**
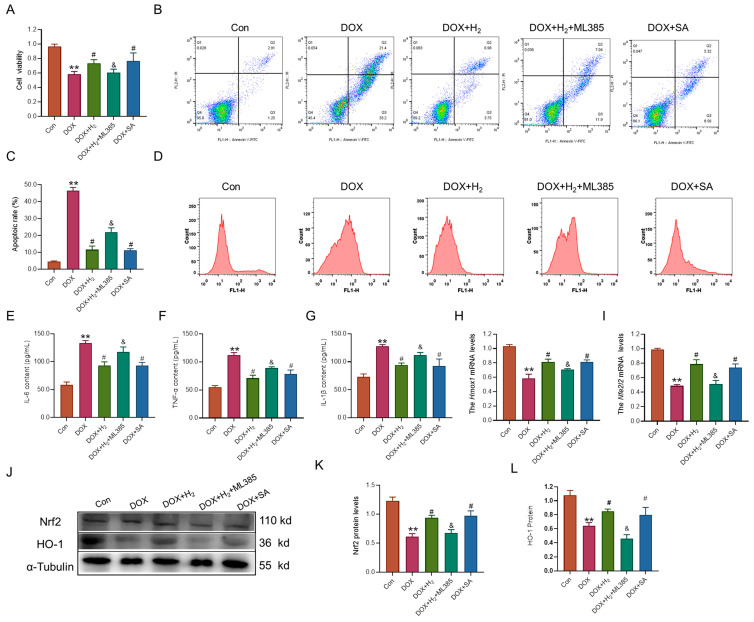
Hydrogen alleviated HepG2 cell damage by regulating the Nrf2 pathway. (**A**) The cell viability of HepG2 cells treated with DOX, hydrogen, ML385, and SA (n = 6). (**B**,**C**) The apoptotic cells in each group using flow cytometry (n = 3). (**D**) The fluorescence absorption spectrum of BODIPY 581/591 C11 using flow cytometry (n = 3). (**E**–**G**) The content of IL-6, IL-1β, and TNF-α in the cell culture medium (n = 3). (**H**,**I**) *Nfe2l2* and *Hmox1* mRNAlevels detected by Q-PCR (n = 3). (**J**–**L**) Nrf2 and HO-1 protein levels detected by western blot (n = 3). The results are presented as the mean ± SEM. ** *p* < 0.01 vs. Con group. ^#^ *p* < 0.05 vs. DOX group, ^&^ *p* < 0.05 vs. DOX+H_2_ group.

To further elucidate the role of hydrogen in mitigating DOX-induced cellular damage, we used HepG2 cells treated with DOX, an Nrf2 agonist (Sappanone A, SA), and an Nrf2 inhibitor (ML385). The results from the CCK8 assay demonstrated a significant reduction in cell viability in the DOX-treated group (Figure 7A). Additionally, flow cytometry analysis indicated an increase in apoptotic cells within the DOX group (Figure 7B,C). Treatment with hydrogen and SA notably improved cell viability and reduced the apoptotic rate. However, the protective effect of hydrogen was attenuated by ML385 (Figure 7A–C). Moreover, the DOX group showed elevated lipid ROS levels, which were significantly reduced following treatment with hydrogen and SA (Figure 7D). ELISA results revealed that the levels of pro-inflammatory cytokines, including IL-6, IL-1β, and TNF-α, were significantly elevated in the culture medium of the DOX-treated group. These cytokine levels were reduced following treatment with hydrogen and SA (Figure 7E–G). Furthermore, Q-PCR and western blot analyses demonstrated that the expression levels of Nrf2 and HO-1 gene and protein were substantially decreased in the DOX-treated groups (Figure 7H–L). Treatment with hydrogen and SA significantly upregulated Nrf2 and HO-1 expression, although the therapeutic effect of hydrogen was again inhibited by ML385.

## 3. Discussion

Cancer continues to be one of the most prevalent diseases affecting humans, with an incidence rate exceeding 440 cases per 100,000 individuals, resulting in approximately 19.3 million new diagnoses annually [17]. DOX, an anthracycline antibiotic, is widely utilized as a chemotherapeutic agent in the treatment of various malignant tumors. However, the administration of high doses or prolonged use of DOX is associated with significant hepatic and cardiac toxicity, often necessitating the discontinuation of chemotherapy or a reduction in dosage [18]. Therefore, managing the toxic side effects of DOX is crucial for the effective treatment of cancer patients.

The molecular mechanism underlying DOX-induced hepatotoxicity is primarily characterized by the generation of ROS during the drug’s hepatic metabolism, leading to a disruption in redox homeostasis [19]. DOX could undergo a reversible oxidation process, resulting in the formation of a semiquinone intermediate, a reaction facilitated by the enzyme nicotinamide adenine dinucleotide phosphate hydrogen (NADPH) reductase [19]. This process results in the production of ROS, including superoxide radicals and hydrogen peroxide, and may also contribute to the upregulation of NAD(P)H quinone dehydrogenase 1 (NQO1) [20]. Concurrently, the generated nitric oxide (NO) could react with either molecular oxygen (O_2_) or superoxide anion (O_2_^−^) to form reactive nitrogen species (RNS), which subsequently induce lipid peroxidation and DNA damage [21]. An increase in the oxidative stress biomarker 8-hydroxy-2′-deoxyguanosine (8-OHdG) has been observed during DOX treatment [22]. Furthermore, the oxidative damage triggers the activation of immune cells, thereby contributing to inflammatory responses. Studies have demonstrated that DOX could exacerbate toxic injury by activating inflammatory pathways, such as the induction of NLRP3-mediated cardiomyocyte pyroptosis [23], increased expression of CD45 and monocyte chemoattractant protein-1 (MCP-1) [22], and enhanced production of pro-inflammatory cytokines in hepatic tissue [24]. Consequently, strategies aimed at reducing oxidative stress may provide a promising avenue for mitigating DOX-induced hepatotoxicity.

Hydrogen serves as a protective gas with significant biological effects, including antioxidant, anti-inflammatory, anti-apoptotic, and anti-ferroptosis properties, and is regarded as a promising regulator of redox homeostasis [25,26,27]. Studies have demonstrated that exogenous hydrogen supplementation could ameliorate various liver injuries, modulate glucose and lipid metabolism in both animal models and humans, and play a crucial role in maintaining liver homeostasis [26,28]. Our findings indicated that hydrogen treatment significantly reduced the levels of ALT, AST, and ALP following DOX administration, thereby alleviating liver tissue damage and inhibiting hepatocyte apoptosis (Figure 1). Hydrogen selectively neutralizes ROS and free radicals, including hydroxyl radicals and peroxynitrite radicals [11,29]. We hypothesized that this protective effect was mediated through the inhibition of oxidative stress. Consistent with this hypothesis, the results showed that DOX significantly decreased oxidative stress levels in liver tissues while enhancing their antioxidant capacity (Figure 4).

Research has shown that hydrogen mitigated liver damage not only through its antioxidant properties but also by inhibiting inflammatory responses [30]. Similar investigations have confirmed that the neuroprotective effects of HRS were associated with both inflammation and oxidative stress during ischemia and reperfusion events [31]. Hydrogen demonstrated potential in reducing inflammatory tissue damage induced by oxidative stress by down-regulating pro-inflammatory cytokines such as IL-1β, IL-6, and TNFα, as well as pro-inflammatory transcription factors like high mobility group box-1 protein (HMGB-1) and NF-κB [32,33]. Consequently, our findings revealed that hydrogen significantly inhibited the inflammatory response induced by DOX, suppressed inflammasome formation, and decreased the expression of inflammatory factors (Figure 5).

**Figure 8:**
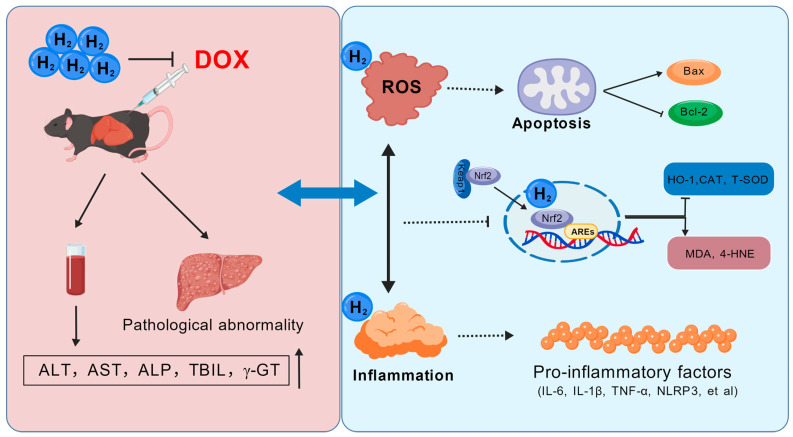
The schematic diagram of hydrogen protection against DOX-induced liver injury. DOX has been observed to provoke biochemical alterations and pathological abnormalities in the liver. Specifically, DOX facilitates the generation of ROS and promotes mitochondria-dependent cell apoptosis. Additionally, DOX impedes the reduction in the expression of Keap1 and Nrf2, thereby inhibiting the downstream antioxidant signaling pathways of Nrf2, including HO-1, CAT, and T-SOD. Concurrently, it enhances the expression of lipid peroxidation products such as MDA and 4-HNE. Furthermore, DOX instigates inflammatory processes and augments the release of pro-inflammatory cytokines. In contrast, hydrogen exerts a protective effect on DOX-induced liver dysfunction by modulating Nrf2, which mitigates oxidative stress and inflammatory responses.

Nrf2 is a transcription factor known for its anti-inflammatory and antioxidant properties [34]. Studies have suggested that hydrogen could modulate the Nrf2 signaling pathway, thereby exerting protective effects such as anti-oxidation, anti-inflammation, and anti-ferroptosis in various disease models [25,27,35]. The specific upregulation of Nrf2 expression has been shown to mitigate DOX-induced liver injury [36]. The Nrf2/HO-1 signaling pathway is considered a promising therapeutic target for metabolic dysfunction-associated steatotic liver disease [37]. However, it remains unclear whether hydrogen could activate Nrf2 to ameliorate DOX-induced liver damage. To address this question, we conducted both in vitro and in vivo experiments. Our findings revealed a reduction in Nrf2 expression in the DOX model, while hydrogen treatment effectively activated Nrf2 expression. In the cellular model, we utilized the Nrf2 inhibitor ML385 and the Nrf2 activator SA [38,39]. The findings indicated that the protective effects of hydrogen on cellular function were partially inhibited by ML385, which impacted both its anti-inflammatory and anti-oxidant properties (Figure 8). Importantly, the Nrf2 activator exhibited effects analogous to those of hydrogen, suggesting that hydrogen may exert its protective effects, at least in part, through the activation of Nrf2 expression. These results suggested that hydrogen may modulate the Nrf2/HO-1 pathway to elicit anti-inflammatory and anti-oxidant responses, thereby mitigating DOX-induced liver toxicity.

Nevertheless, this study is constrained by specific limitations. Chiefly, the experiments were conducted solely in vitro to assess the impact of hydrogen on Nrf2. Future research should include in vivo studies to offer a more comprehensive understanding. Furthermore, while the regulation of Nrf2 by hydrogen was observed, the precise mechanisms underlying this regulation within the organism require further investigation.

## 4. Materials and Methods

### 4.1. Animals

C57BL/6N male mice, with body weights ranging from 22 g to 24 g and classified as specific pathogen-free (SPF), were obtained from Henan Skobes Biotechnology Co., Ltd., located in Anyang, China. The mice were housed in the laboratory animal center under controlled temperature and humidity conditions. All procedures involving animal handling and experimentation complied with the National Guidelines for the Management and Use of Laboratory Animals and were approved by the Ethical Committee for Animal Experiments at Hebei University of Chinese Medicine (ethics approval no. KJLL(D) 20251026).

A total of 40 C57BL/6N mice were randomly assigned to four groups: the control group (Con), the DOX group, the DOX combined with hydrogen-rich saline solution (DOX+HRS) group, and the control combined with hydrogen-rich saline solution (Con+HRS) group. Mice in the DOX and DOX+HRS groups received intraperitoneal injections of the DOX solution at a dosage of 4 mg/kg, administered four times over a four-week period. The mice in the DOX+HRS and Con+HRS groups were administered an HRS solution at a dosage of 10 mL/kg/day via intraperitoneal injection. The control groups received the same volume of normal saline.

After the experiment concluded, the body weights of the mice in each group were measured. Following anesthesia administration using tribromoethanol (200 mg/kg), tissue samples from the liver and spleen were collected. The liver and spleen tissues were then determined, and the liver index and spleen index were calculated (the respective tissue weights/body weight).

### 4.2. Preparation of HRS

The HRS was prepared in accordance with the manufacturer’s guidelines (HuiMei Medical Technology, Shanghai, China). In summary, hydrogen was dissolved in normal saline for six hours at 0.4 Megapascals (MPa) to achieve a supersaturated state using a hydrogen production apparatus. The HRS was freshly prepared each day to sustain a concentration of 0.6 mmol/L. A needle-type hydrogen sensor (Unisense A/S) was employed to monitor the hydrogen concentration [27,40].

### 4.3. Reagents and Antibodies

Reagents: Nrf2 inhibitor ML385 (HY-100523, MedChemExpress, Shanghai, China), Nrf2 agonist Sappanone A (SA, HY-113556, MedChemExpress, Shanghai, China), T-SOD (A001-1, Nanjing Jiancheng Bioengineering Institute, Nanjing, China), MDA (A003-1, Nanjing Jiancheng Bioengineering Institute, Nanjing, China), CAT (A007-1, Nanjing Jiancheng Bioengineering Institute, Nanjing, China), mouse/human IL-6 ELISA kit (E-EL-M0044/E-EL-H6156, Elabscience, Wuhan, China), mouse/human TNF-α (EK0527/EK0525, Boster, Wuhan, China), mouse IL-1β (EK0394/EK0392, Boster, Wuhan, China), RNA extraction kit (DP419, Tiangen Biotech, Beijing, China), PrimeScript™ RT regent Kit with gDNA Eraser (RR047A, Takara, Dalian, China), SYBR-Green PCR Master Mix kit (RR820A, Takara, Dalian, China), Tunel kit (G1507, Servicebio, Wuhan, China).

Antibodies: Bcl-2 (GB153375, Servicebio, Wuhan, China), Bax (GB11007, Servicebio, Wuhan, China), Caspase 3 (#14220, Cell Signaling Technology, Danvers, MA, USA), MDA (ab243066, Abcam, Cincinnati, OH, USA), 4-HNE (ARG23717, Arigo Biolaboratories, Hsinchu, Taiwan), IL-6 (DF6087, Affinity, Cincinnati, OH, USA), NLRP3 (BA3677, Boster, Wuhan, China), Nrf2 (GB115673, Servicebio, Wuhan, China), HO-1 (GB12104, Servicebio, Wuhan, China), Keap1 (bs-4900R, BIOSS, Shanghai, China), and α-Tubulin (GB11200, Servicebio, Wuhan, China).

### 4.4. Biochemical Analysis

Colorimetric assays were used to detect biochemical markers in serum to assess liver function. The levels of ALT, AST, ALP, TBIL, andγ-GT were measured using a fully automated biochemistry analyzer (BK-400, Biobase Biodustry, Jinan, China) in accordance with the manufacturer’s specifications.

### 4.5. Pathological Staining

Hematoxylin and eosin (HE) staining was performed to examine the fundamental architecture of liver tissue. Paraffin-embedded liver sections (5 µm) were first dewaxed in xylene and rehydrated through a graded alcohol series. Subsequently, the sections were stained with hematoxylin, differentiated in hydrochloric acid ethanol, counterstained with eosin, dehydrated through graded alcohol, vitrified in xylene, and, finally, mounted with neutral gum.

Masson’s trichrome staining was employed to assess hepatic fibrosis. Following deparaffinization, the sections were sequentially treated with hematoxylin, hydrochloric acid ethanol, Masson blue solution, distilled water, ponceau red, phosphomolybdic acid, and aniline blue. They were then dehydrated in ethanol, vitrified in xylene, and mounted with neutral gum.

Sirius Red staining was also utilized to evaluate liver fibrosis. After deparaffinization and rehydration, the sections were stained with Sirius Red, dehydrated in ethanol, vitrified in xylene, and mounted with neutral medium. Images were acquired using a microscope.

### 4.6. Transmission Electron Microscope

A transmission electron microscope (TEM) was used to observe the ultrastructure of mitochondria. After inducing deep anesthesia, liver tissue was promptly isolated and dissected into fragments not exceeding 1 mm^3^. Post-fixation, the tissue specimens were dehydrated using a graded ethanol series and embedded in Araldite. Ultrathin sections were subsequently stained with 4% uranyl acetate and imaged using a TEM (HT7800, Hitachi, Tokyo, Japan).

### 4.7. GEO Data Analysis

The microarray dataset GSE11940 was obtained from the GEO repository (https://www.ncbi.nlm.nih.gov/geo/, accessed on 1 September 2025). The raw data files, formatted in MINiML, were downloaded and subsequently processed. Background correction and quantile normalization were conducted using R software (version 4.4.3), with probes being mapped to gene symbols. In instances where multiple probes corresponded to the same gene, their values were averaged. Differentially expressed genes (DEGs) were identified utilizing the Limma package (version 3.60.3) in R software (version 4.4.3), applying thresholds of |log2 fold change (FC)| > 1 and an adjusted *p* < 0.05. A Volcano plot was generated to illustrate the distribution of DEGs. Enrichment analyses for GO categories—cellular component (CC), molecular function (MF), and biological process (BP)—and KEGG pathways were performed on the DEGs using the clusterProfiler package (version 4.14.6) in R (version 4.4.3), with an adjusted *p* < 0.05 set as the criterion for significant enrichment. The results were visualized using a Circular plot for GO terms and a Bubble plot for KEGG pathways. Expression levels of *IL-6*, *NLRP3*, *NOX4*, *SOD2*, and *Nfe2l2* were extracted, and Box plots were employed to compare expression differences between groups.

### 4.8. Immunological Staining

IHC was used to examine protein expression in liver tissue. Paraffin-free sections were incubated with 3% hydrogen peroxide to eliminate endogenous peroxidases. Antigen retrieval was subsequently performed at high temperatures using a citrate antigen retrieval buffer (10 mM, pH 6.0). The sections were then blocked with 10% goat serum for 1 h at 37 °C. Following this, the liver sections were incubated overnight at 4 °C with primary antibodies targeting 4-HNE, IL-6, Nrf2, and HO-1. The following day, the sections were incubated with horseradish peroxidase (HRP)-conjugated secondary antibodies for 1 h at 37 °C. The sections were then enhanced and stained using a DAB kit (ZLI-9018, Zhongshan Jinqiao Biotechnology Co., Ltd., Beijing, China). After sealing the sections with neutral resin, images were captured, and the mean density of positive cells was quantified using Image-Pro Plus (IPP) software (version 6.0; Media Cybernetics, Rockville, MD, USA).

### 4.9. Tunel Staining

Tunel staining was employed to evaluate apoptosis in hepatic tissue. Following dewaxing and rehydration, the tissue sections were rinsed with phosphate-buffered saline (PBS, 0.01 M, pH 7.4). Subsequently, a proteinase K solution (20 μg/mL) was applied for 20 min at room temperature (RT). After PBS washing, the sections were incubated with a reaction mixture containing a bright green labeling mixture and recombinant TdT enzyme at 37 °C for 60 min. Thereafter, streptavidin-HRP was added and incubated at 37 °C for 60 min. The sections were then enhanced and stained using a DAB kit. Finally, the sections were sealed with neutral resin, images were captured, and the mean density of positive cells was quantified utilizing IPP software (version 6.0; Media Cybernetics, Rockville, MD, USA).

### 4.10. Q-PCR Analysis

Quantitative PCR (Q-PCR) was employed to assess the expression of *Nfe2l2* (Nrf2 gene) and *Hmox1* (HO-1) mRNA. Total RNA was extracted from liver tissue using an RNA extraction kit (DP419, Tiangen Biochemical Technology Co., Ltd., Beijing, China). Subsequently, 1 μg of the extracted RNA was reverse-transcribed into cDNA utilizing the PrimeScript™ RT Reagent Kit with the gDNA Eraser. Q-PCR amplification was conducted with the SYBR-Green PCR Master Mix kit. The expression levels of Nfe2l2 and Hmox1 mRNA were quantified using a Q-PCR instrument (CFX Connect, Bio-Rad Laboratories, Hercules, CA, USA). The primer sequences employed for Q-PCR are listed in Table 1. Relative gene expression was calculated using the 2^−ΔΔCt^ method.

### 4.11. Western Blot Analysis

Initially, pre-cooled RIPA lysate was added to liver tissue or HepG2 cells, followed by homogenization and centrifugation. Protein concentration was subsequently determined using the bicinchoninic acid (BCA) assay. Thereafter, proteins were resolved by sodium dodecyl sulfate–polyacrylamide gel electrophoresis (SDS-PAGE) and transferred onto polyvinylidene fluoride (PVDF) membranes. The membranes were then blocked with 5% skim milk powder and incubated overnight at 4 °C with primary antibodies targeting Bax, Bcl-2, Caspase 3, MDA, 4-HNE, NLRP3, IL-6, Keap1, Nrf2, HO-1, and α-Tubulin. On the following day, the membranes were incubated with HRP-conjugated secondary antibodies for 2 h at RT. Finally, immunoreactive proteins were detected using the enhanced chemiluminescence (ECL) method, and the mean gray value of the immunoreactive bands was quantified using Image J software (version 1.8.1, NIH, Bethesda, MD, USA).

### 4.12. Cell Culture and Viability Assay

HepG2 cells were cultured in Dulbecco’s Modified Eagle Medium (DMEM) supplemented with 10% fetal bovine serum (FBS), penicillin, and streptomycin at 37 °C in an atmosphere containing 5% CO_2_. A cellular model of DOX exposure was established at a concentration of 5 μM for 24 h. During DOX treatment, cells in the H_2_ group were subjected to a hydrogen-rich medium, prepared analogously to HRS.

Cell viability was assessed using the Cell Counting Kit-8 (CCK-8, K1018, ApeBio, Houston, TX, USA). HepG2 cells were seeded at a density of 1 × 10^4^ cells per well in 96-well culture plates. Following treatment with or without DOX, hydrogen (H_2_), ML385 (2 μM), and SA (20 μM), the CCK-8 reagent was added to the wells and incubated at 37 °C for 1 h. Absorbance was subsequently measured at 450 nm using a multifunctional microplate reader (Varioskan LUX, Thermo Fisher Scientific, Waltham, MA, USA).

### 4.13. ELISA Analysis

Inflammatory cytokines in serum and cell culture medium were assessed by ELISA. Briefly, serum and cell culture supernatants were collected, and the concentrations of IL-6, IL-1β, and TNF-α were quantified according to the manufacturer’s instructions. Standard curves were generated for each cytokine, and absorbance was measured at 450 nm to determine the expression levels of these inflammatory factors.

### 4.14. Statistical Analysis

The experimental data were analyzed using SPSS statistical software, version 26.0. The measurement data underwent the Shapiro–Wilk test to assess normality and the Levene test to evaluate homogeneity of variance. Data with normal distribution and homogeneous variance were expressed as mean ± standard error of the mean (SEM) and subjected to statistical analysis using one-way analysis of variance (ANOVA) followed by the least significant difference (LSD) post hoc test. Conversely, data not conforming to normal distribution or displaying heterogeneous variance were examined using the Kruskal–Wallis H test. A significance level was set at *p* ≤ 0.05. Statistical visualizations were produced using Prism software, version 9.0.

## 5. Conclusions

In conclusion, this study suggests that DOX may induce hepatotoxicity through the enhancement of oxidative stress and inflammatory responses. Conversely, hydrogen appeared to confer anti-inflammatory and anti-oxidant benefits by upregulating the expression of Nrf2, thereby alleviating the DOX-induced liver toxicity. These findings offer a theoretical basis for the clinical application of hydrogen.

## Figures and Tables

**Table 1:** The sequence of primers used for the expression of genes.

Gene	Forward	Reverse
*β-actin*	AGGCCCAGAGCAAGAGAGGTA	TCTCCATGTCGTCCCAGTTG
*Nfe2l2*	GTCACATCGAGAGCCCAGTC	TGGCTTCTGGACTTGGAACC
*Hmox1*	ACATGCCTATACACGCTATCTCG	CGTCACTCCAGGAAATGAGAAGA

## Data Availability

The data presented in this study are available on request from the corresponding author (Y.-S.Z.).

## Abbreviations

The following abbreviations are used in this manuscript:

4-HNE	4-Hydroxynonenal
8-OHdG	8-hydroxy-2′-deoxyguanosine
ALP	Alkaline phosphatase
ALT	Alanine transaminase
AST	Aspartate transaminase
BCA	Bicinchoninic acid
CAT	Catalase
CCK-8	Cell Counting Kit-8
DMEM	Dulbecco’s Modified Eagle Medium
DOX	Doxorubicin
ELISA	Enzyme-linked immunosorbent assay
ECL	Enhanced chemiluminescence
FBS	Fetal bovine serum
GEO	Gene Expression Omnibus
H_2_	Hydrogen
HE	Hematoxylin and eosin
HMGB-1	High-mobility group box-1 protein
HO-1	Heme oxygenase-1
HRS	Hydrogen-rich saline solution
IHC	Immunohistochemical staining
IL-1β	Interleukin-1β
IL-6	Interleukin-6
IPP	Image-Pro Plus
MCP-1	Chemoattractant protein-1
MDA	Malondialdehyde
NADPH	Enzyme nicotinamide adenine dinucleotide phosphate hydrogen
NLRP3	NOD-like receptor protein 3
NO	Nitric oxide
NQO1	NAD(P)H quinone dehydrogenase 1
Nrf2	Nuclear factor erythroid-derived 2-like 2
PBS	Phosphate-buffered saline
PVDF	Polyvinylidene fluoride
Q-PCR	Quantitative PCR
RNS	Reactive nitrogen species
